# Reproducibility in PD-L1 Immunohistochemistry Quantification through the Tumor Proportion Score and the Combined Positive Score: Could Dual Immunostaining Help Pathologists?

**DOI:** 10.3390/cancers15102768

**Published:** 2023-05-16

**Authors:** Anaïs Mercier, Virginie Conan-Charlet, Isabelle Quintin-Roué, Laurent Doucet, Pascale Marcorelles, Arnaud Uguen

**Affiliations:** 1 CHU de Brest, Service D’anatomie et Cytologie Pathologiques, F-29200 Brest, France; anais.mercier@chu-brest.fr (A.M.); virginie.conan-charlet@chu-brest.fr (V.C.-C.); isabelle.quintin-roue@chu-brest.fr (I.Q.-R.); laurent.doucet@chu-brest.fr (L.D.); pascale.marcorelles@chu-brest.fr (P.M.); 2 LBAI, UMR1227, Inserm, CHU de Brest, Univ Brest, F-29200 Brest, France

**Keywords:** PD-L1, immunohistochemisry, TPS, CPS, reproducibility

## Abstract

**Simple Summary:**

The quantification of PD-L1 expression in tumor samples through the tumor proportion score (TPS) and the combined positive score (CPS) conditions provides access to anti-PD-1/PD-L1 immunotherapy in patients with various solids cancers. Reproducibility studies have shown very heterogeneous inter- and intra-pathologist agreements, from poor to excellent ones, in TPS and CPS quantification. We studied the inter- and intra-pathologist agreements in TPS and CPS quantification comparing single PD-L1 immunohistochemistry (S-IHC) and double immunohistochemistry (D-IHC) combining PD-L1 staining and tumor nuclear markers, trying to improve the distinction between tumor cells and immune cells necessary to TPS and CPS calculations. Our study concluded in excellent (TPS) to good (CPS) inter- and intra-pathologist agreements with both S-IHC and D-IHC with slightly higher intraclass correlation coefficients using D-IHC. D-IHC could help the pathologists to quantify PD-L1 expression through TPS and CPS for subsequent therapeutic choices in patients with advanced cancers.

**Abstract:**

We studied the pathologists’ agreements in quantifying PD-L1 expression through the tumor proportion score (TPS) and the combined positive score (CPS) using single PD-L1 immunohistochemistry (S-IHC) and double immunohistochemistry (D-IHC) combining PD-L1 staining and tumor cell markers. S-IHC and D-IHC were applied to 15 cancer samples to generate 60 digital IHC slides (30 whole slides images and 30 regions of interest of 1 mm^2^) for PD-L1 expression quantification using both TPS and CPS, twice by four pathologists. Agreements were estimated calculating intraclass correlation coefficients (ICC). Both S-IHC and D-IHC slides analyses resulted in excellent (for TPS, ICC > 0.9) to good (for CPS, ICC > 0.75) inter- and intra-pathologist agreements with slightly higher ICC with D-IHC than with S-IHC. S-IHC resulted in higher TPS and CPS than D-IHC (+5.6 and +6.1 mean differences, respectively). High reproducibility in the quantification of PD-L1 expression is attainable using S-IHC and D-IHC.

## 1. Introduction

Targeting the PD-1/PD-L1 axis using immune checkpoint inhibitors (ICIs) is now approved to treat patients with different advanced solid cancers. Nevertheless, highly different responses to ICIs are observed in patients in terms of antitumor responses, from no response to complete remissions, but also in terms of immune-related adverse effects simulating autoimmune diseases in some patients [1]. To better stratify patients and anticipate their response to ICIs, predictive strategies have been proposed, such as the quantification of PD-L1 expression using immunohistochemistry (IHC) on tumor samples.

For some PD-1/PD-L1 ICIs, as a companion diagnostic, PD-L1 IHC conditions the possibility to use the treatment itself or provide its use in a first-line of treatment if the tumor sample sufficiently expresses the PD-L1 molecule. In other cases, it only consists of a complementary test of predictive and/or prognostic significance but does not condition the use of the treatment. For both these predictive and prognostic purposes, the guidelines for the quantifications of PD-L1 expression vary from one cancer to another. Indeed, PD-L1 expression could be quantified in the tumor cells (TC) solely leading to a “tumor proportion score” (TPS) expressed in terms of percentage of PD-L1-positive TC (with complete or incomplete membranous staining of any intensity) or in association with the expression by mononuclear immune cells (IC) except plasma cells (i.e., lymphocytes and monocytes–macrophages, with membranous and/or cytoplasmic IHC staining) through a “combined positive score” (CPS). In this manner, both the quantifications of TPS and CPS require distinguishing between TC and IC, PD-L1-positive, and PD-L1-negative, which could sometimes be difficult. In addition to various TPS or CPS quantification methods, positivity cut-off values vary from one cancer to another, but also from one ICI to another. Different PD-L1 IHC clones, protocols, and automates can also produce variations in IHC signals. Adding the intra- and intertumor spatial and time heterogeneity of PD-L1 expression, there are several potential difficulties and sources of discrepancies in the scoring of PD-L1 expression [2].

Another potential issue in PD-L1 expression quantification could also be inter- and intra-pathologists’ agreements in PD-L1 IHC quantification [3,4,5,6,7,8,9,10,11,12,13,14,15]. The evaluation of the reproducibility in assessing PD-L1 TPS and CPS using single PD-L1 IHC (S-IHC) but also using a more original dual IHC (D-IHC) method combining PD-L1 IHC with tumor nuclear marker IHC is the aim of the present study.

## 2. Material and Methods

### 2.1. Cases Selection

The cases included in this study were selected among patients with cancer samples (melanomas and NSCLC) tested for PD-L1 expression for diagnostic purpose in the Department of Pathology of CHU Brest. S-IHC and D-IHC had been performed on the same tissue block for each case and were collected from archives as well as corresponding hematoxylin–eosin–saffron (HES) slides to select a panel of 15 cases of cancers (1) of different organs and histological subtypes (6 melanomas and 9 NSCLC samples, see Table 1 for summary), and (2) with different levels of PD-L1 IHC expression according to initial pathology reports from no to diffuse staining. Cases were deidentified for this reproducibility study. Clinical data about treatment choices and responses to treatments of the patients were not collected in this study, which was conducted in accordance with our national and institutional guidelines, in compliance with the Helsinki Declaration, and after approval by our institutional review board (CHRU Brest, CPP n° DC—2008—214).

### 2.2. Immunohistochemistry

The clone 22C3 (1:50 dilution; Dako, Glostrup, Denmark) was the anti-PD-L1 antibody used for IHC in the present study. The S-IHC analyses were performed on tissue sections, 3 μm thick, laid on Superfrost Plus slides using the Ventana Benchmark Ultra automated slide preparation system (Roche Diagnostics, Meylan, France) and OptiView DAB IHC Detection Kit (Roche Diagnostics). A PD-L1 positive control (tonsil) was added on each IHC slide. The S-IHC staining procedure included a pretreatment step with cell conditioner 1, followed by incubation with the anti-PD-L1 diluted antibody at 37 °C. Antibody incubation and signal amplification was followed by counterstaining with hematoxylin, washing, and mounting. For D-IHC, the slides underwent an antibody denaturation step at 95 °C for 8 min after incubation with the PD-L1 antibody revealed in DAB, as described for S-IHC slides, and before the incubation with a second antibody, targeting a tumor nuclear marker and revealed in red using the ultraView Universal Alkaline Phosphatase Red Detection Kit (Roche Diagnostics). The nuclear markers used in our study were TTF-1 (clone 8G7G3/1, 1:50 dilution, Dako, 64 min CC1 pretreatment), p40 (polyclonal, 1:100 dilution, Clinisciences (Nanterre, France), 36 min CC1 pretreatment), and p53 (clone DO-7, 1:50 dilution, Dako, 64 min CC1 pretreatment) for NSCLC samples and SOX10 (clone SP267, prediluted, Cell-Marque, 64 min CC1 pretreatment) for melanoma samples. After the incubation of the second antibody, the slide was counterstained with hematoxylin, washed, and mounted.

### 2.3. Slides Digitalization

HES, S-IHC, and D-IHC were digitalized using a 3DHistech Pannoramic Midi scanner (3Dhistech, Budapest, Hungary) at ×40 magnification resulting in MRXS-files whole slides images (WSIs). The CaseViewer software (3Dhistech) was used to visualize the WSIs and select, through the built-in annotation tool, small particular tissue regions of interest (ROIs) of 1 mm^2^ (1 per IHC slide) on the basis of different PD-L1 staining abundance on the S-IHC slide, from no to diffuse staining with intermediate proportions of stained cells. The same ROI selected on the S-IHC slide was selected on the corresponding D-IHC slide. Selected ROIs were then exported separately of their native WSIs into new MRXS files using the Pannoramic Viewer software (3DHistech). Once the whole WSIs and ROIs MRXS had been produced, the whole set of MRXS files was duplicated into two image sets (set 1 and set 2) and slide labels were changed between the two image sets to prevent cross-identification by observers.

### 2.4. PD-L1 Expression Quantification

**Figure 1:**
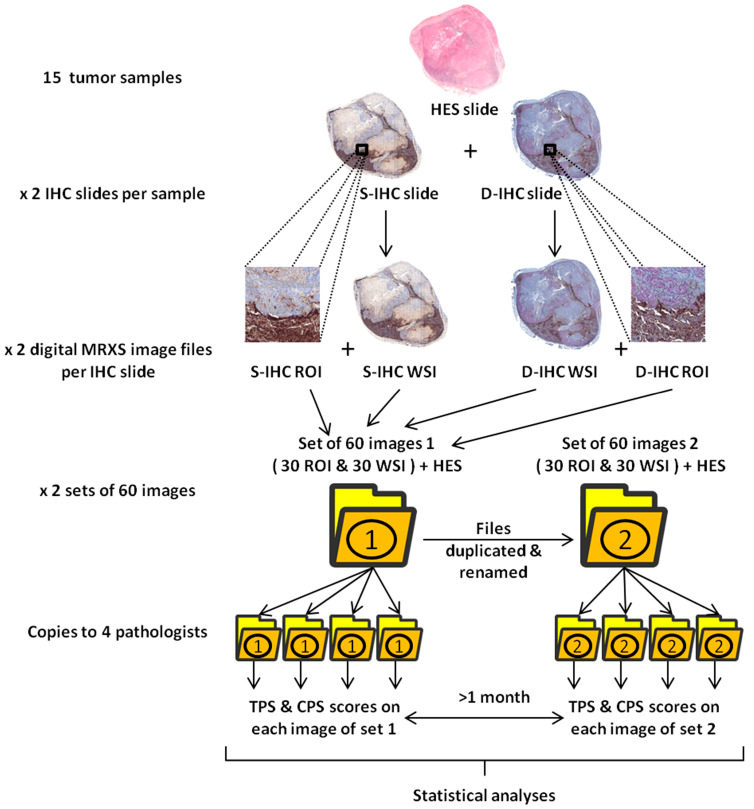
Summary of study design. HES: hematoxylin–eosin–saffron; IHC: immunohistochemistry; S-IHC: single PD-L1 immunohistochemistry slide; D-IHC: dual immunohistochemistry slide combining PD-L1 and tumor nuclear markers; ROI: 1 mm^2^ selected region of interest; WSI: whole slide image; TPS: tumor proportion score; CPS: combined positive score.

PD-L1 expression was quantified by four pathologists. Each pathologist analyzed the whole image sets of ROI and WSI files following both the TPS and the CPS criteria. Each pathologist performed two analyses of each slide through the separated interpretations of set 1 and set 2 images with at least 1 month between the analyses of the two images sets. For each IHC images, the corresponding HES images were also provided to the pathologists. The study design is summarized in Figure 1.

The percentage of TC with a membranous staining (complete or incomplete, of any intensity) evaluated on the whole MRXS image was used for the calculation of the TPS. CPS was defined as the number of PD-L1-stained cells (TC with membrane staining; IC cells as lymphocytes and macrophages with membranous and/or cytoplasmic staining) divided by the total number of viable TC, multiplied by 100. Necrotic areas were not taken into account for these quantifications. For each observer and each analysis, exact count values of TPS and CPS were collected.

### 2.5. Statistical Analyses

Statistical analyses were performed using MedCalc Statistical Software version 13.2.2 (MedCalc Software, Ostend, Belgium). Intra-class correlations coefficients (ICCs) were used to estimate the inter- and intra-observer agreements for TPS and CPS and were interpreted as follows: <0.5: poor reliability, 0.5–0.75: moderate reliability, 0.75–0.9: good reliability, >0.9: excellent reliability. Bland–Altman plots were used to represent the differences between the two IHC methods, and scatter diagrams were used to illustrate the standard deviations between the measurements of the four pathologists as a function of the means of their rating for each image.

## 3. Results

### 3.1. Intra- and Inter-Pathologist Reproducibility in TPS Quantification

The global agreement between the four pathologists and for each pathologist was globally excellent for TPS scoring (i.e., ICC > 0.9), for WSI and ROI images, with nevertheless more issues in the analysis of melanoma samples (resulting in poor inter-pathologist agreement and a moderate intra-pathologist agreement) in comparison with NSCLC samples (excellent intra- and inter-pathologist agreements). The melanin pigmentation in tumor cells and melanophages, sometimes difficult to differentiate from DAB-brown staining could contribute to explaining the inferior performances in melanoma samples compared to in NSCLC ones. The intra- and inter-pathologist agreements were excellent using either S-IHC or D-IHC, with slight trends of higher ICC using D-IHC in comparison with S-IHC (see Table 2 for detailed values).

### 3.2. Intra- and Inter-Pathologist Reproducibility in CPS Quantification

The global agreement between the four pathologists and for each pathologist was globally good for CPS scoring (i.e., ICC between 0.75 and 0.9), with the lowest ICC and intra- and inter-pathologist agreements in analyzing the melanoma samples as reported for the TPS scoring. Of note, the CPS calculated on WSI resulted in higher intra- and inter-pathologist agreement (said excellent through ICC interpretation) in comparison with those calculated on the basis of ROI. Most of the ICCs calculated for TPS and CPS resulted in lower values and inferior intra- and inter-pathologist agreements for CPS values than for TPS ones. With regard to the TPS, there were slight trends of higher ICC using D-IHC in comparison with S-IHC, but both IHC methods resulted in good intra- and inter-pathologist agreements (see Table 2 for detailed values).

### 3.3. Differences in TPS and CPS between S-IHC and D-IHC Methods

**Figure 2:**
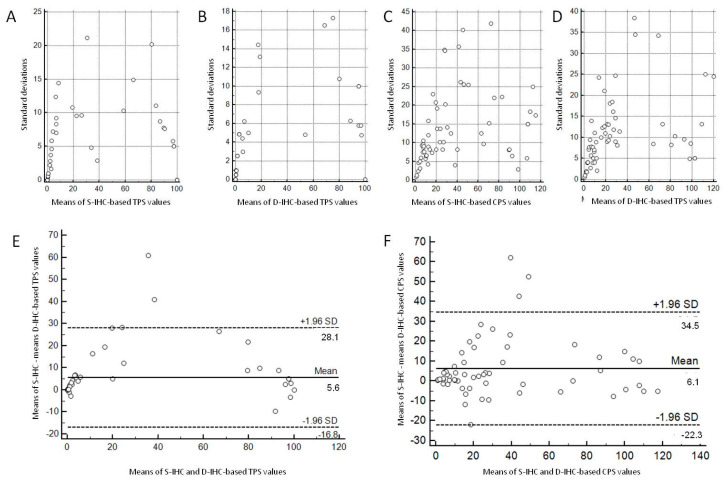
Diagrams summarizing the distributions of PD-L1 immunohistochemistry values. Inter-observer values are illustrated using scatter diagrams of measurements standard deviations as function of means of measurements by the four pathologists (**A**–**D**), and differences between the two methods are illustrated using Bland–Altman plots (**E**,**F**). IHC: immunohistochemistry; S-IHC: single PD-L1 immunohistochemistry slide; D-IHC: dual immunohistochemistry slide combining PD-L1 and tumor nuclear markers; TPS: tumor proportion score; CPS: combined positive score.

**Figure 3:**
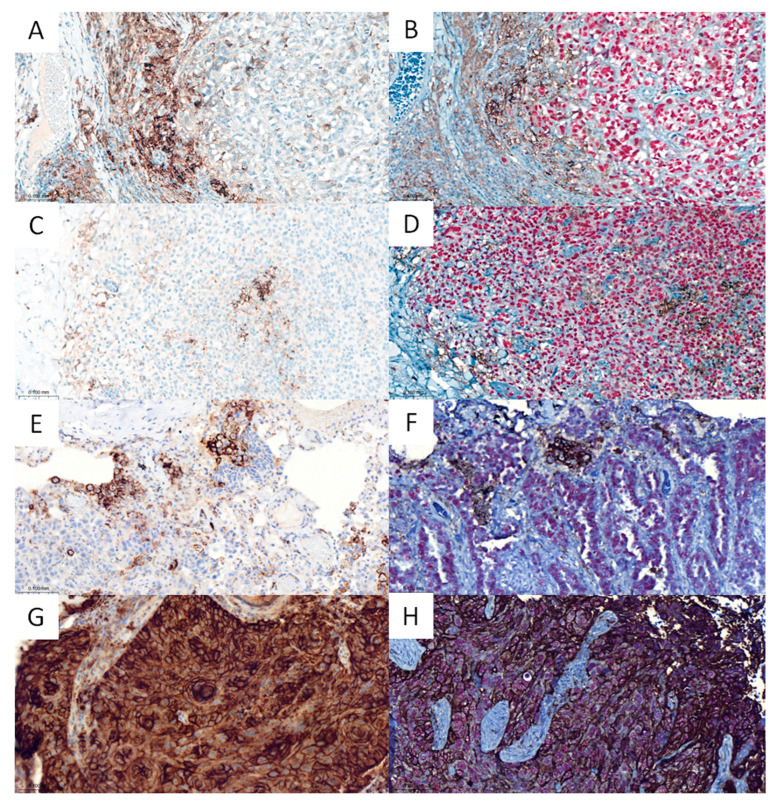
Examples of PD-L1 immunohistochemistry results obtained in the same regions per cases using single PD-L1 immunohistochemistry (S-IHC, (**A**,**E**,**G**)) and dual immunohistochemistry slide combining PD-L1 and tumor nuclear markers (D-IHC, (**B**,**D**,**F**,**H**)). (**A**,**B**) superficial spreading melanoma; (**C**–**F**) lung adenocarcinomas; (**G**,**H**) lung squamous cell carcinoma; anti-PD-L1 immunohistochemistry appears in DAB-brown staining and nuclear markers appear in red staining; (**B**) anti-SOX10, (**D**) anti-TTF-1, (**F**) anti-p53, (**H**) anti-p40; all pictures are at ×200 magnification. In images (**A**–**F**), PD-L1-positive are only immune cells whereas in images (**G**,**H**), tumor cells are diffusely PD-L1-positive.

Scatter diagrams illustrating the standard deviations as function of the means of TPS and CPS data measured by the four pathologists indicated a trend that higher standard deviations were obtained in cases with TPS and CPS values far from 0 and 100, indicating more inter-pathologist discrepancy results in these cases. The same trend was observed for both S-IHC and D-IHC. The Bland–Altman plots method was concordant with this trend and also pointed out that the TPS and CPS calculated on the basis of D-IHC tended to be inferior to those calculated on the basis of S-IHC (mean differences of 5.6 for TPS and 6.1 for CPS, differences being inferior around the scores of 0 and 100 and more pronounced far from these values). See Figure 2 for the graphs and Figure 3 for images of paired S-IHC and D-IHC.

## 4. Discussion

Several biomarkers can condition the access to anti-PD-1/PD-L1 ICIs in patients with different advanced cancers such as tumor mutational burden, microsatellite instability, and, more recently, promising mature tertiary lymphoid structures identification [16,17,18]. PD-L1 IHC expression quantification using either TPS or CPS scores with various positivity cut-offs from one cancer type to another is also one of these biomarkers and has been the subject of several reproducibility studies in the literature. These reproducibility studies are also very heterogeneous in terms of anti-PD-L1 IHC clones and platforms, the natures of cancers, the number of cases and pathologists, the use of physical glass slides or digital ones, the need for exact quantification of positive cells, or the use of semiquantitative scales and different statistical methods. Inter- and intra-observer reproducibility is reported with different agreements, from poor to excellent ones, but all the aforementioned methodological variations make the different studies difficult to compare [3,4,5,6,7,8,9,10,11,12,13,14,15].

Of note, despite the interest and potential issues in differentiating TC and IC in tumor samples to calculate TPS and CPS, studies investigating the potential interest of coupling anti-PD-L1 IHC with TC nuclear markers using D-IHC were lacking in the literature. Thus, we chose to focus the present reproducibility study on this D-IHC method and its comparison with the S-IHC one. In our study, intra- and inter-pathologist reproducibility was globally good to excellent, with notably better results in assessing TPS than in assessing CPS, highlighting more issues in quantifying PD-L1 expression in IC and TC than in TC only. Although ICC interpretation conclusions were similar between S-IHC and D-IHC, ICC values were slightly higher with D-IHC than with S-IHC, pointing out the potential interest of D-IHC as an aid in differentiating between PD-L1-positive TC and IC. Comparisons of S-IHC and D-IHC also revealed that S-IHC TPS and CPS scores were higher than D-IHC ones, pointing out a potential reclassification from PD-L1-positive TC on S-IHC images to PD-L1-positive IC ones using D-IHC. Because the use of D-IHC tends to result in inferior PD-L1 scores compared to in S-IHC, this could have consequences in terms of therapeutic decisions for tumor samples with scores below the currently established thresholds of “PD-L1 positivity” (established only on the basis of S-IHC to date) according to TPS and/or CPS scores in various cancers. This has to be kept in mind for pathologists using D-IHC as a complementary analysis to avoid reclassification of patients candidates to ICI treatments from a “PD-L1 positive” status to a “PD-L1-negative” status.

The variations between analyses were also higher when the mean proportions of PD-L1 cells were far from the lowest and highest ones, pointing out particular potential issues for pathologists in the case of heterogeneous IHC staining. There is no doubt that in a near future, novel artificial-intelligence-based software applied to digital pathology slides could be of great help to pathologists in analyzing those heterogeneous cases, allowing them to gain in reproducibility [19,20,21,22].

## 5. Conclusions

High intra- and inter-pathologist agreement is attainable in scoring PD-L1 expression through the TPS and CPS immunohistochemistry scores. In cases with difficult-to-differentiate tumor and immune cells, D-IHC can help pathologists to better calculate these scores, conditioning the access of patients to ICI treatments.

## Figures and Tables

**Table 1:** Summary of the 15 studied tumor samples (all surgical specimens, no biopsy). IHC: immunohistochemistry.

6 Melanomas	9 NSCLC
6 primary cutaneous tumors	5 primary lung tumors 4 metastases (2 nodal, 2 cerebral)
4 superficial spreading melanomas IHC anti-SOX10 diffusely positive 2 nodular melanomas IHC anti-SOX10 diffusely positive	3 adenocarcinomas IHC anti-TTF-1 diffusely positive 2 adenocarcinomas IHC anti-p53 IHC diffusely positive (IHC anti-TTF-1 only focally positive, not taken into account in the present study) 3 squamous cell carcinomas IHC anti-p40 diffusely positive 1 squamous cell carcinomas IHC anti-p53 diffusely positive (IHC anti-p40 only focally positive, not taken into account in the present study)

**Table 2:** Inter- and intra-pathologist agreement according to intraclass correlation coefficient (ICC) calculations (ICC values; 95% confidence intervals, and interpretation of the reliability). TPS: tumor proportion score; CPS: combined positive score; WSI: whole slide image; NSCLC: non-small-cell lung carcinomas; S-IHC: single PD-L1 immunohistochemistry slide; D-IHC: dual immunohistochemistry slide combining PD-L1 and tumor nuclear markers.

Inter-Pathologist Reproducibility	TPS	CPS
Global	0.9702 [0.9602; 0.9792] Excellent agreement	0.8202 [0.7700; 0.8636] Good agreement
Images set 1	0.9751 [0.9625; 0.9842] Excellent agreement	0.8308 [0.7603; 0.8878] Good agreement
Images set 2	0.9652 [0.9485; 0.9776] Excellent agreement	0.8089 [0.7320; 0.8718] Good agreement
Melanoma	0.07982 [−0.02977; 0.2292] Poor agreement	0.1974 [0.07088; 0.3562] Poor agreement
NSCLC	0.9669 [0.9520; 0.9781] Excellent agreement	0.7807 [0.7028; 0.8467] Good agreement
WSI	0.9716 [0.9578; 0.9818] Excellent agreement	0.9007 [0.8530; 0.9362] Excellent agreement
ROI	0.9686 [0.9525; 0.9801] Excellent agreement	0.7238 [0.6238; 0.8104] Moderate agreement
S-IHC	0.9652 [0.9466; 0.9782] Excellent agreement	0.7913 [0.7089; 0.8596] Good agreement
D-IHC	0.9759 [0.9642; 0.9846] Excellent agreement	0.8519 [0.7894; 0.9020] Good agreement
**Intra-Pathologist Reproducibility**	**TPS**	**CPS**
Global	0.9797 [0.9737; 0.9843] Excellent agreement	0.8543 [0.8151; 0.8857] Good agreement
Pathologist #1	0.9789 [0.9641; 0.9876] Excellent agreement	0.8531 [0.7522; 0.9128] Good agreement
Pathologist #2	0.9844 [0.9737; 0.9907] Excellent agreement	0.8975 [0.8345; 0.9374] Good agreement
Pathologist #3	0.9923 [0.9871; 0.9954] Excellent agreement	0.8675 [0.7876; 0.9187] Good agreement
Pathologist #4	0.9564 [0.9264; 0.9744] Excellent agreement	0.7717 [0.6385; 0.8601] Good agreement
Melanoma	0.6216 [0.4805; 0.7312] Moderate agreement	0.5672 [0.4147; 0.6889] Moderate agreement
NSCLC	0.9756 [0.9659; 0.9825] Excellent agreement	0.8028 [0.7288; 0.8573] Good agreement
WSI	0.9812 [0.9731; 0.9869] Excellent agreement	0.9319 [0.9033; 0.9522] Excellent agreement
ROI	0.9781 [0.9682; 0.9849] Excellent agreement	0.77 [0.6841; 0.8347] Good agreement
S-IHC	0.9779 [0.9684; 0.9846] Excellent agreement	0.8268 [0.76; 0.8763] Good agreement
D-IHC	0.9816 [0.9735; 0.9872] Excellent agreement	0.8840 [0.8352; 0.9187] Good agreement

## Data Availability

Data available upon reasonable request to the corresponding author.
